# Novel genes associated with enhanced motility of *Escherichia coli* ST131

**DOI:** 10.1371/journal.pone.0176290

**Published:** 2017-05-10

**Authors:** Asha Kakkanat, Minh-Duy Phan, Alvin W. Lo, Scott A. Beatson, Mark A. Schembri

**Affiliations:** 1 School of Chemistry and Molecular Biosciences, University of Queensland, Brisbane, Queensland, Australia; 2 Australian Infectious Disease Research Centre, University of Queensland, Brisbane, Queensland, Australia; 3 Australian Centre for Ecogenomics, University of Queensland, Brisbane, Queensland, Australia; Imperial College London, UNITED KINGDOM

## Abstract

Uropathogenic *Escherichia coli* (UPEC) is the cause of ~75% of all urinary tract infections (UTIs) and is increasingly associated with multidrug resistance. This includes UPEC strains from the recently emerged and globally disseminated sequence type 131 (ST131), which is now the dominant fluoroquinolone-resistant UPEC clone worldwide. Most ST131 strains are motile and produce H4-type flagella. Here, we applied a combination of saturated Tn*5* mutagenesis and transposon directed insertion site sequencing (TraDIS) as a high throughput genetic screen and identified 30 genes associated with enhanced motility of the reference ST131 strain EC958. This included 12 genes that repress motility of *E*. *coli* K-12, four of which (*lrhA*, *ihfA*, *ydiV*, *lrp*) were confirmed in EC958. Other genes represented novel factors that impact motility, and we focused our investigation on characterisation of the *mprA*, *hemK* and *yjeA* genes. Mutation of each of these genes in EC958 led to increased transcription of flagellar genes (*flhD* and *fliC*), increased expression of the FliC flagellin, enhanced flagella synthesis and a hyper-motile phenotype. Complementation restored all of these properties to wild-type level. We also identified Tn*5* insertions in several intergenic regions (IGRs) on the EC958 chromosome that were associated with enhanced motility; this included *flhDC* and EC958_1546. In both of these cases, the Tn*5* insertions were associated with increased transcription of the downstream gene(s), which resulted in enhanced motility. The EC958_1546 gene encodes a phage protein with similarity to esterase/deacetylase enzymes involved in the hydrolysis of sialic acid derivatives found in human mucus. We showed that over-expression of EC958_1546 led to enhanced motility of EC958 as well as the UPEC strains CFT073 and UTI89, demonstrating its activity affects the motility of different UPEC strains. Overall, this study has identified and characterised a number of novel factors associated with enhanced UPEC motility.

## Introduction

Uropathogenic *Escherichia coli* (UPEC) are the most common cause of urinary tract infection (UTI), a disease of major significance to global human health [1–3]. UPEC employ a range of virulence factors to colonise the urinary tract and cause symptomatic UTI, including adhesins, toxins, iron-acquisition systems, polysaccharide surface structures and flagella [4–8]. Overall, the combined affect of genetic variation, redundancy and genomic diversity means that no single virulence factor is uniquely associated with the ability of UPEC to cause disease. This complex picture is further convoluted by increased resistance to antibiotics, which complicates the treatment of UTI and highlights the urgent need to better understand UPEC pathogenesis. A major contributor to increased antibiotic resistance among UPEC is the fluoroquinolone-resistant sequence type 131 (ST131) clone, which has emerged recently and disseminated rapidly across the globe [9–11].

Flagella are complex multi-subunit, filamentous organelles that contribute to various aspects of UPEC virulence, including motility, chemotaxis, adhesion, biofilm formation and immune modulation [5, 12–14]. In mice, flagella provide a fitness advantage for UPEC colonization of the urinary tract, leading to increased colonization and persistence in mixed competitive infection experiments comprising wild-type and isogenic flagella mutant strains [15, 16]. Flagella-mediated motility is also required for UPEC ascension to the upper urinary tract and subsequent dissemination to other sites [17]. Complementing these studies, others have shown that flagella also contribute to UPEC invasion of mouse renal epithelial collecting duct cells [5] and enhanced adhesion to and invasion of bladder epithelial cells [14]. Flagella are also required for UPEC biofilm formation on abiotic surfaces [12].

The biosynthesis, assembly and regulation of *E*. *coli* flagella have been the subject of extensive research over many decades [18–21]. The flagella structure contains three distinct components, the basal body, hook and an extracellular filament composed of the major subunit protein FliC or flagellin. The FliC is highly immunogenic and sequence variation within its hyper-variable central region defines the *E*. *coli* H antigen diagnostic serotype marker [22]. The synthesis and assembly of flagella occurs via a highly ordered process that involves a combination of transcriptional, translational and post-translational regulatory mechanisms. At the transcriptional level, the regulation of flagella is coordinated via a hierarchical cascade that involves three stages of control [23]; the FlhDC master regulators control the first stage of this process. Numerous global regulatory proteins influence flagella expression by either positively or negatively regulating the transcription of *flhDC* [24, 25]. Major transcriptional activators of *flhDC* include the cyclic AMP-catabolite activator protein (CRP) [26], the histone-like nucleoid-structuring (H-NS) protein [26, 27], the quorum sensing *E*. *coli* regulators B and C (QseBC) [28–30] and the MatA regulator of the *E*. *coli* common pilus [31]. Conversely, major transcriptional repressors of *flhDC* include the LysR-type regulator LrhA [32], the osmoregulator protein OmpR [33], the colanic acid activator Rcs [34], the P fimbriae-associated regulator PapX [35, 36], the ferric uptake regulatory protein (Fur) [37] and integration host factor (IHF) [38]. Mutation of these regulatory genes alters the transcription of *flhDC* and leads to either reduced or enhanced motility.

Our understanding of *E*. *coli* motility has been enhanced by the application of large-scale genetic screens to study flagella expression and chemotaxis. Overall, these studies have shown that many different cell processes influence this complex phenotype. For example, Girgis *et al*. (2007) performed a powerful genome-wide investigation that combined competitive selection and microarray analysis, and resulted in the characterization of thirty-six novel motility-associated genes [39]. These genes encoded for a diverse range of non-flagellar factors, and notably comprised a large number of cell envelope proteins including transporters, periplasmic enzymes and intrinsic membrane proteins. Another study by Inoue *et al*. (2007) screened a comprehensive collection of *E*. *coli* K-12 mutants (the Keio collection) and compiled a detailed compendium of genes involved in swimming and swarming motility [40]. Again, a range of non-flagellar genes were identified, including those encoding factors associated with metabolism, iron acquisition, protein-folding and the biosynthesis of lipopolysaccharide (LPS) as well as other cell-surface components. Large-scale genetic screens to study motility in *Salmonella* have also been performed, with similar classes of genes identified [41, 42]. Interestingly, a set of genes associated with enhanced motility (hyper-motility) of *Salmonella* were identified in one of these studies; in some cases this phenotype was associated with increased expression of flagellin on the cell surface [41].

While the flagella regulon from *E*. *coli* has been extensively studied, the identification and characterisation of genes associated with hyper-motility has not been examined in great detail. We recently described the combined application of saturated Tn*5* mutagenesis and transposon directed insertion site sequencing (TraDIS) to comprehensively define the complete set of genes associated with resistance to human serum in the UPEC ST131 strain EC958 [43]. Here, we applied TraDIS as a large scale genetic screen and identified a series of genes that when mutated, led to increased motility of the UPEC ST131 reference strain EC958.

## Results

### Identification of genes associated with enhanced motility of EC958

**Fig 1:**
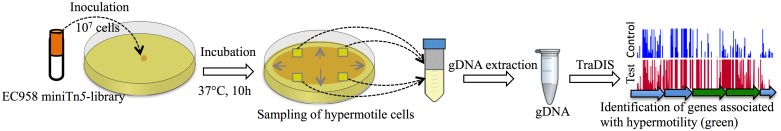
Schematic describing the experimental strategy to identify genes in EC958 associated with hyper-motility. Approximately 1x10^7^ Tn*5* mutants (input pool) were spotted in the center of 20 LB agar (0.25%) plates and incubated at 37°C. Motile cells were recovered by extracting LB agar at the edge of the swimming zone (output pool). EC958 genomic DNA was purified from both pools and analysed by TraDIS.

We devised a swimming assay in combination with a forward genetic screen using a previously generated hyper-saturated mini-Tn*5* mutant library [43] to identify genes associated with enhanced motility of EC958 (Fig 1). In this assay, a pool of approximately 1x10^7^ Tn*5* mutants (input pool) was spotted in the center of 20 soft LB agar plates and incubated for 10 hours at 37°C. Motile cells were recovered from the edge of the swimming zone of each plate by extracting the LB agar at a distance of 30mm from the point of inoculation (output pool). EC958 genomic DNA was purified from the input and output pools and sequenced using a multiplexed TraDIS procedure. The input and output pools yielded 8.4x10^5^–7x10^6^ Tn*5*-specific reads, 78% of which could be mapped to the EC958 genome (S1 Table).

Using a stringent threshold cutoff (log_2_ fold change [logFC] > 5; *P* < 0.001), 30 genes were identified that, when mutated, led to enhanced motility of EC958 (Table 1). For each of these genes, the number of reads corresponding to Tn*5* insertions was significantly increased in cells at the periphery of the swimming zone (output pool) compared to the input pool. Twelve of these genes have previously been shown to repress motility, namely *lrhA* [32], *ihfA* [38], *ydiV* [44, 45], *ihfB* [38] *lrp* [46], *clpXP* [47], *papX* [35, 36], *rcsB* [34] *pfrA* [48], *yeaI* [49] and *fliT* [50]. The remaining genes represent novel factors that influence the motility of EC958, with their function ranging across eight Clusters of Orthologous Groups (COGs) (Table 1).

**Table 1:** Genes identified by TraDIS in the motility screen.

EC958 locus_tag	Gene Name	Matched in MG1655	logFC	Product	COG code*
EC958_2624	*lrhA*^#^	b2289	10.45	transcriptional repressor of flagellar, motility genes	K
EC958_0577	*clpX*^#^	b0438	9.85	ATP-dependent protease Clp	O
EC958_1934	*ihfA*^#^	b1712	9.51	integration host factor (IHF)	L
EC958_1480	*hemK*	b1212	9.48	N5-glutamine methyltransferase	R
EC958_3401	*yghB*	b3009	9.17	general envelope maintenance protein	S
EC958_4909	*ydfD*	b1576	9.15		-
EC958_1929	*ydiV*^#^	b0688	8.88	anti-FlhD_4_C_2_ factor	T
EC958_2115	*yecG*	b1895	8.82	universal stress protein	T
EC958_1122	*ihfB*^#^	b0912	8.78	integration host factor (IHF) beta subunit	L
EC958_3259	*papX*^#^	b2684	8.76	fimbrial repressor of flagellar Synthesis	K
EC958_4635	*yjeK*	b4146	8.72	EF-P-Lys34 lysylation protein	E
EC958_0576	*clpP*^#^	b0437	8.68	protease subunit of ATP-dependent serine protease	O
EC958_1001	*lrp*^#^	b0889	8.63	DNA-binding transcriptional dual regulator	K
EC958_4646	*yjeA*	b4155	8.11	Elongation Factor P Lys34 lysyltransferase	J
EC958_2149	2149	b1548	7.86	Phage DNA packaging protein	X
EC958_2136	2136	b2373	7.72	Phage related tail fiber protein	X
EC958_2299	*yeeL*	b4497	7.46	ADP-heptose—LPS heptosyltransferase 2	M
EC958_2949	*mprA*	b2684	6.90	MarR-like transcriptional regulator	K
EC958_4172	*gyrB*	b3699	6.60	DNA gyrase, subunit B	J
EC958_2554	*rcsB*^#^	b2217	6.60	Capsular biosynthesis protein	T
EC958_2007	*yeaI*^#^	b1785	5.83	predicted membrane-anchored diguanylate cyclase	T
EC958_0185	*apaH*	b0049	5.74	diadenosine tetraphosphatase	T
EC958_2194	*fliT*^#^	b1926	5.67	Regulates the transcription of class 2 flagellar operons	-
EC958_2015	*yeaP*	-	5.59	diguanylate cyclase	T
EC958_1479	*prfA*^#^	b1211	5.57	peptide chain release factor RF-1	J
EC958_2767	*eutA*	b2451	5.36	reactivating factor for ethanolamine ammonia lyase	E
EC958_4902	4902	-	5.28		-
EC958_1930	*nlpC*	b1708	5.28	Endopeptidase, P60 family, Cell wall-associated hydrolase	M
EC958_4230	*yifE*	b3764	5.23	Uncharacterized conserved protein	S
EC958_0600	*tomB*	b0461	5.04	Hha toxicity attenuator	-

^#^ Genes previously known to be involved in repression of motility

*COG codes: K-Transcription, O- Posttranslational modifications, protein turn over, chaperons, E- Amino acid transport and metabolism, L-Replication, recombination and repair, R-General function prediction only, S-Function unknown, T-Signal transduction, J- Translation, ribosomal structure and biogenesis, M-Cell wall, membrane, envelope biogenesis.

### Genetic characterisation of selected hyper-motility mutants

**Fig 2:**
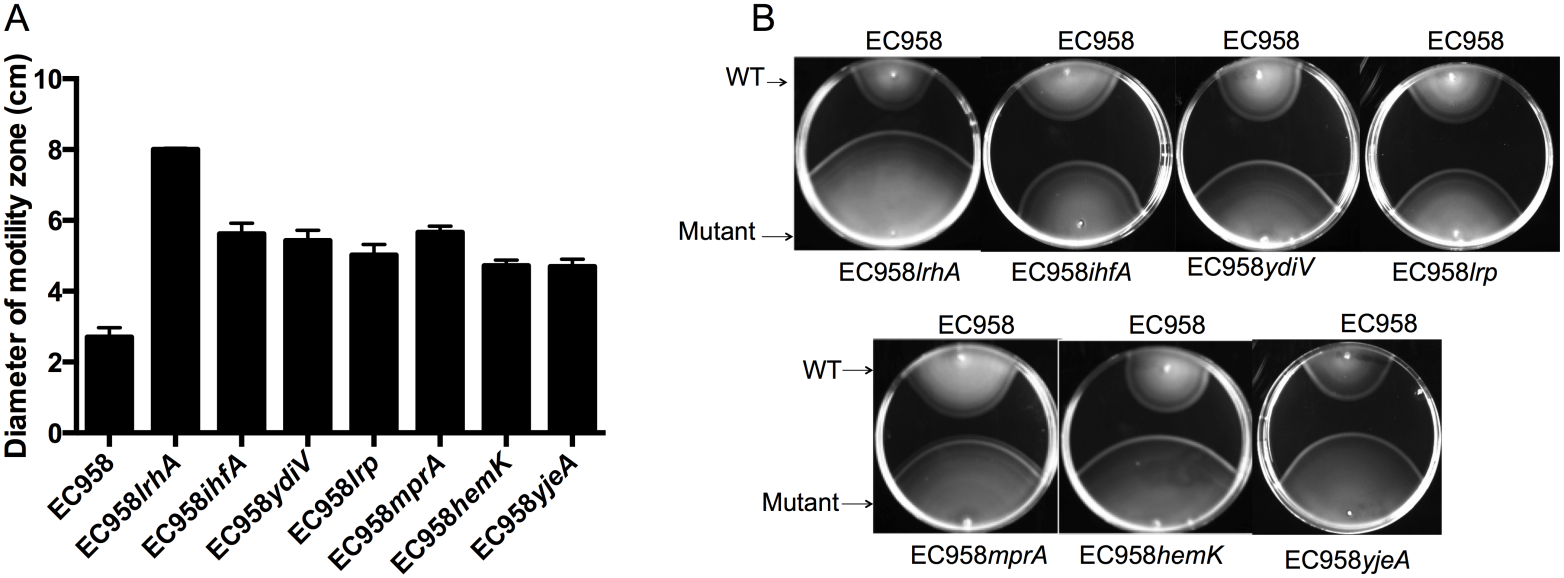
Motility phenotypes of EC958 hyper-motility mutants. (A) The diameter of the swimming zone from EC958 (wild-type) and mutant strains was measured after 7 hours of incubation at 37°C on 0.25% LB agar. The results are based on three replicates; error bars indicate standard deviations. (B) Comparative motility of the same wild-type and mutant strains in 0.25% LB agar. The results from a single experiment, representative of three independent experiments, are shown.

**Fig 3:**
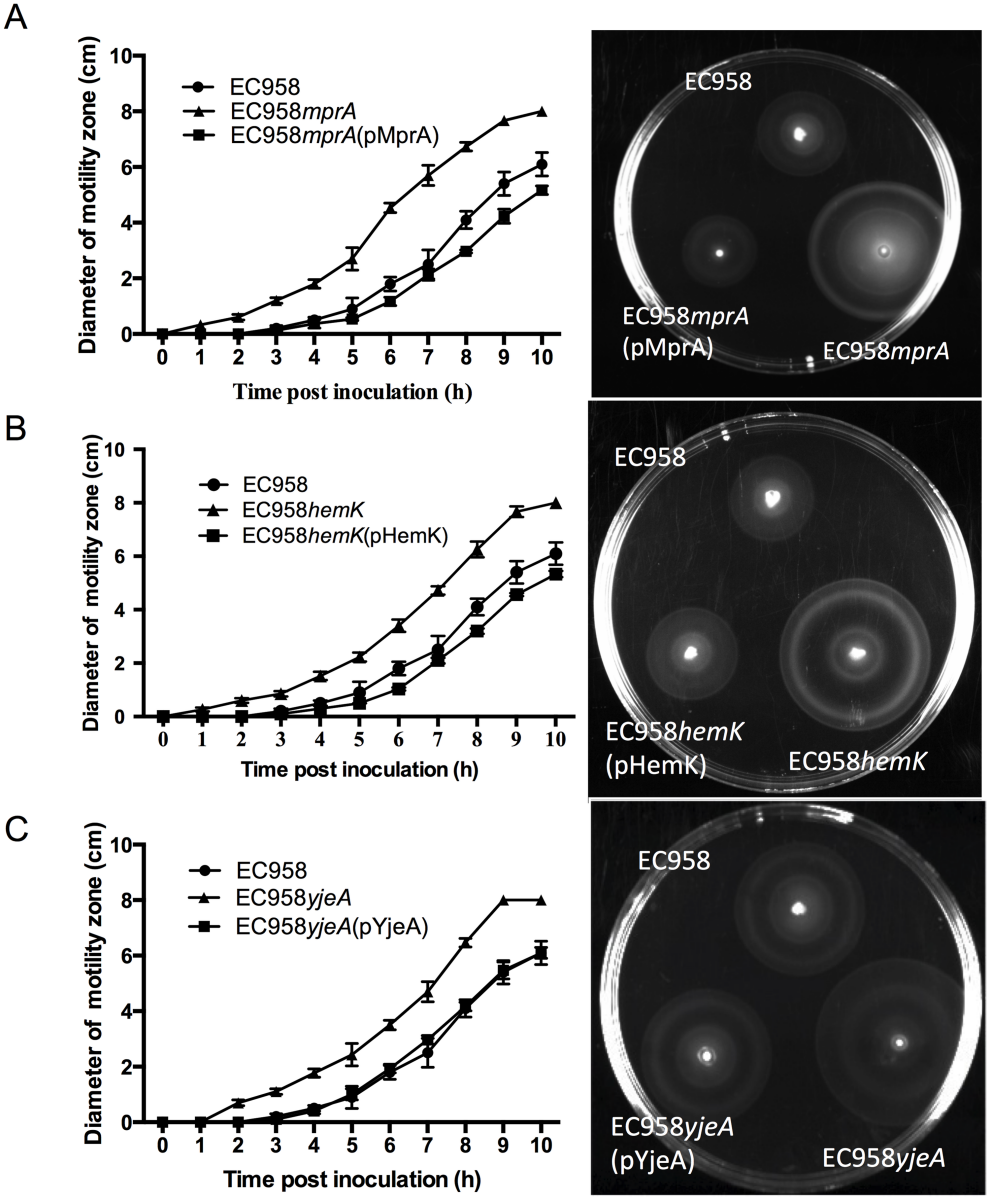
Motility of the (A) EC958mprA, (B) EC958hemK, (C) EC958yjeA mutants and their respective parent and complemented strains. Left panels, rate of motility expressed as the mean diameter of the swimming zone per hour for each strain (+/-standard deviation; n = 3). Right panels, representative motility plates comparing the swimming phenotype of wild-type, mutant and complemented strains.

In order to extend our TraDIS data we validated a selection of the genes involved in repression of motility by generating targeted mutants for further investigation. Thus, EC958 mutants containing deletions in four genes previously shown to repress motility in *E*. *coli* K-12 (*lrhA*, *ihfA*, *ydiV*, *lrp*) and three novel motility-associated genes (*mprA*, *hemK*, *yjeA*) were constructed by λ Red-mediated recombination and characterized in motility assays. In these experiments, all seven mutants displayed an enhanced swimming phenotype on 0.25% LB agar compared to the wild-type EC958 strain (Fig 2). To further confirm the role of the novel motility-associated *mprA*, *hemK* and *yjeA* genes, the genes were cloned in the low copy number plasmid pSU2718G and introduced into their respective mutants to enable genetic complementation. The wild-type, mutant and complemented strains were then grown on 0.25% LB agar to compare their swimming phenotype. In each case, the motility rate of the complemented mutants was restored to wild-type level (Fig 3). Taken together, this data confirms the involvement of *mprA*, *hemK* and *yjeA* in EC958 motility and suggests the TraDIS analysis has accurately identified a collection of genes that when mutated, lead to enhanced motility of EC958.

### Mutation of *mprA*, *hemK* and *yjeA* enhances the transcription and translation of flagella genes

**Fig 4:**
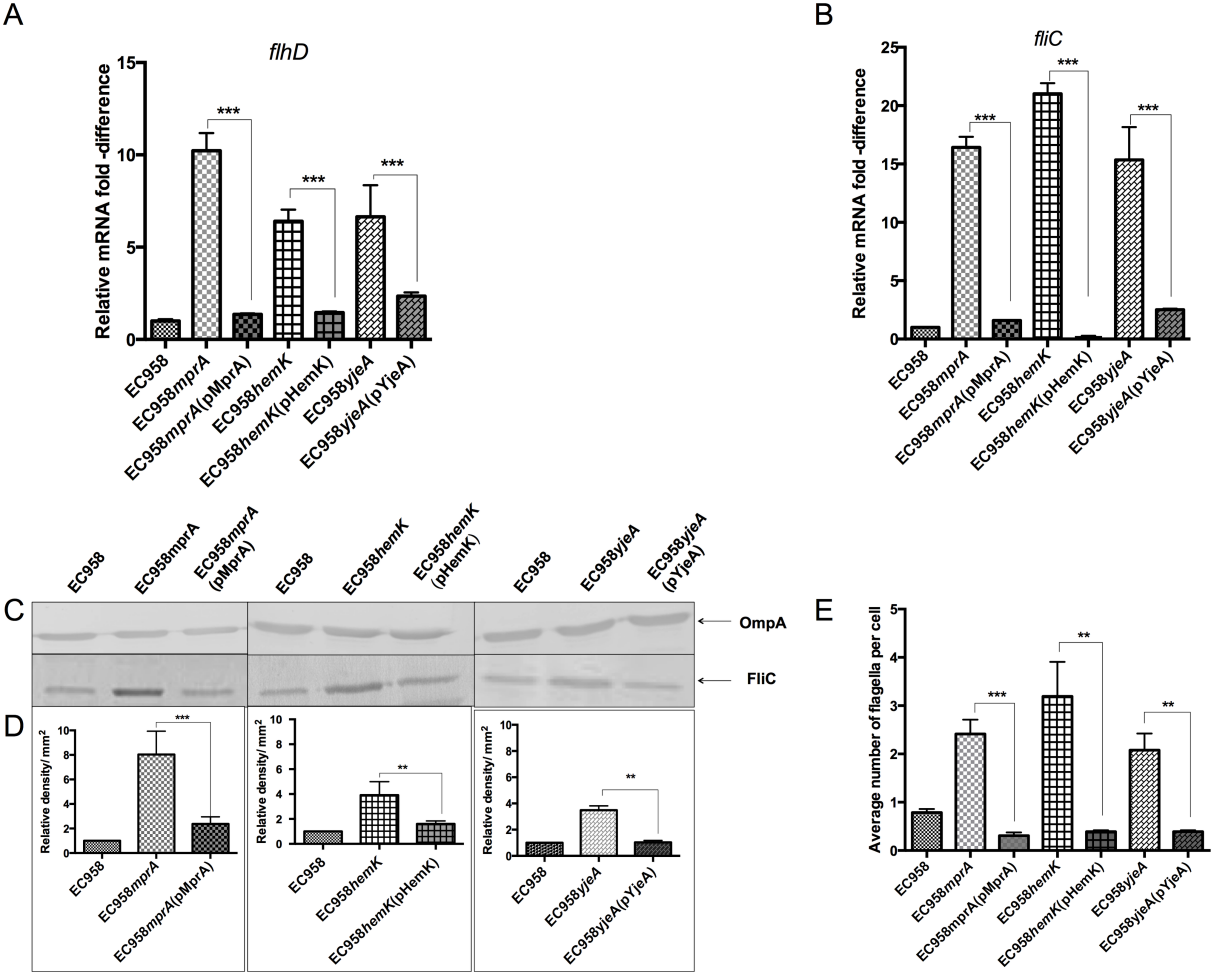
Mutation of *mprA*, *hemK* and *yjeA* leads to increased flagellar gene transcription, flagellin expression and flagella production. (A and B) Quantitative RT-PCR demonstrating the transcription level of (A) *flhD* and (B) *fliC* genes in wild-type, mutant and complemented strains grown to mid-log phase. (C) Western blot analysis of cell lysates prepared from the same mid-log phase culture of each strain probed with an antibody against the H4 FliC flagellin protein (bottom panel) and OmpA (loading control; top panel). (D) Quantitative densitometry analysis of FliC protein production. (E) Average number of flagella counted from 200 cells for each strain following visualization by TEM; the mean and standard deviation from three independent experiments are shown. **P<0.01; ***P<0.001.

To further understand the mechanism by which mutation of *mprA*, *hemK* and *yjeA* could enhance motility, we analysed our set of wild-type, mutant and complemented strains by examining (i) transcription of the *flhD* and *fliC* genes, (ii) expression of the FliC flagellin protein, and (iii) the number of flagella on the cell surface. Mutation of the *mprA*, *hemK* and *yjeA* genes led to a significant increase in the transcription of *flhD* (10.2, 6.4 and 6.6 fold increase, respectively) and *fliC* (16.4, 21.0, 15.2 fold increase, respectively), while complementation of the mutants restored the transcript of *flhD* and *fliC* to wild-type levels (Fig 4A and 4B). In line with these data, the levels of FliC flagellin observed by western blotting using an H4-specific antibody were also elevated in all three mutants compared to the wild-type and complemented strains (Fig 4C and 4D). To link these elevated levels of flagella biosynthesis to the number of flagella organelles per cell, transmission electron microscopy was employed. Based on a count of 200 randomly selected cells for each strain, wild-type EC958 had an average of 0.8±0.1 flagella per cell, which of interest was relatively low compared to strains used for routine studies of motility and chemotaxis [51]. In contrast, the three mutants all possessed significantly higher numbers of flagella per cell; EC958*mprA* 2.4±0.3 flagella/cell, EC958*hemK* 3.3±0.7 flagella/cell and EC958*yjeA* 2.0±0.4 flagella/cell. Complementation of each of the mutants reduced the average number of flagella per cell back to wild-type level (Fig 4E, S1 Fig). Taken together, our data suggest these three genes play a role in controlling the number of flagella per cell and disruption of any of the genes results in hyper-motility by increasing the number of flagella on the cell surface.

### Transposon insertions in intergenic regions associated with enhanced motility

The very high level of saturation in our miniTn*5* library enabled us to compare the insertion frequency within intergenic regions (IGRs) between input and output pools, and thus determine the impact of insertions in these regions on hyper-motility. There are 3973 IGRs on the chromosome of EC958, eight of which contained significantly more miniTn*5* insertions in the output pools than in the input pools. Out of these eight IGRs, six were located upstream of coding sequences (CDS) which are known to repress motility and were discovered in our primary TraDIS analysis (Table 2). The orientation of the miniTn*5* cassette within these six IGRs was unidirectional, such that the chloramphenicol resistance gene was orientated in the opposite direction of the downstream genes and thus the insertion most likely abolished their transcription. The identification of an increased miniTn*5* insertion frequency in both the promoter region and CDS of these genes provides further evidence to support the conclusion that their disruption leads to a hyper-motile phenotype.

**Table 2:** Intergenic regions identified by TraDIS in the motility screen.

EC958 locus_tag	Affected gene by miniTn*5* insertion	Gene name	logFC	FDR	COG code
EC958_IGR1610	EC958_2114	flhD*	10.28	1.28E-26	
EC958_IGR0370	EC958_0577	*clpX*^#^	9.79	4.67E-21	O
EC958_IGR2028	EC958_2624	*lrhA*^#^	9.27	2.42E-22	K
EC958_IGR1456	EC958_1929	*ydiV*^#^	8.58	7.81E-16	T
EC958_IGR1146	EC958_1546	1546*	8.47	9.77E-16	
EC958_IGR0812	EC958_1122	*ihfB*^#^	8.17	3.17E-13	L
EC958_IGR0723	EC958_1001	*lrp*^#^	7.55	4.60E-17	K
EC958_IGR0369	EC958_0576	*clpP*^#^	7.26	4.49E-14	O

Chloramphenicol promoter of miniTn5 transposons were inserted in the * same direction of gene,

^#^ opposite direction of gene

The two remaining IGRs identified by TraDIS (EC958_IGR1610, upstream of *flhD* and EC958_IGR1146, upstream of EC958_1546) contained miniTn5 insertions uniquely located such that the chloramphenicol resistance gene was orientated in the same direction as the downstream gene. Furthermore, in both cases, the downstream gene was devoid of miniTn*5* insertions, suggesting that the function of these genes was required for motility and that their increased transcription (via read-through from the chloramphenicol resistance gene promoter in the miniTn*5* transposon) could result in hyper-motility. In the case of insertions in the IGR upstream of *flhDC*, this interpretation is consistent with other literature that has shown overexpression of these master regulator genes leads to hyper-motility [52–57]. However, we also confirmed this by introducing a strong constitutive promoter (PcL) upstream of the *flhDC* CDS to generate EC958PcL*flhDC*; as expected, this strain exhibited enhanced motility and produced more FliC protein than wild-type EC958 (S2 Fig).

### Overexpression of the EC958_1546 gene enhances EC958 motility

**Fig 5:**
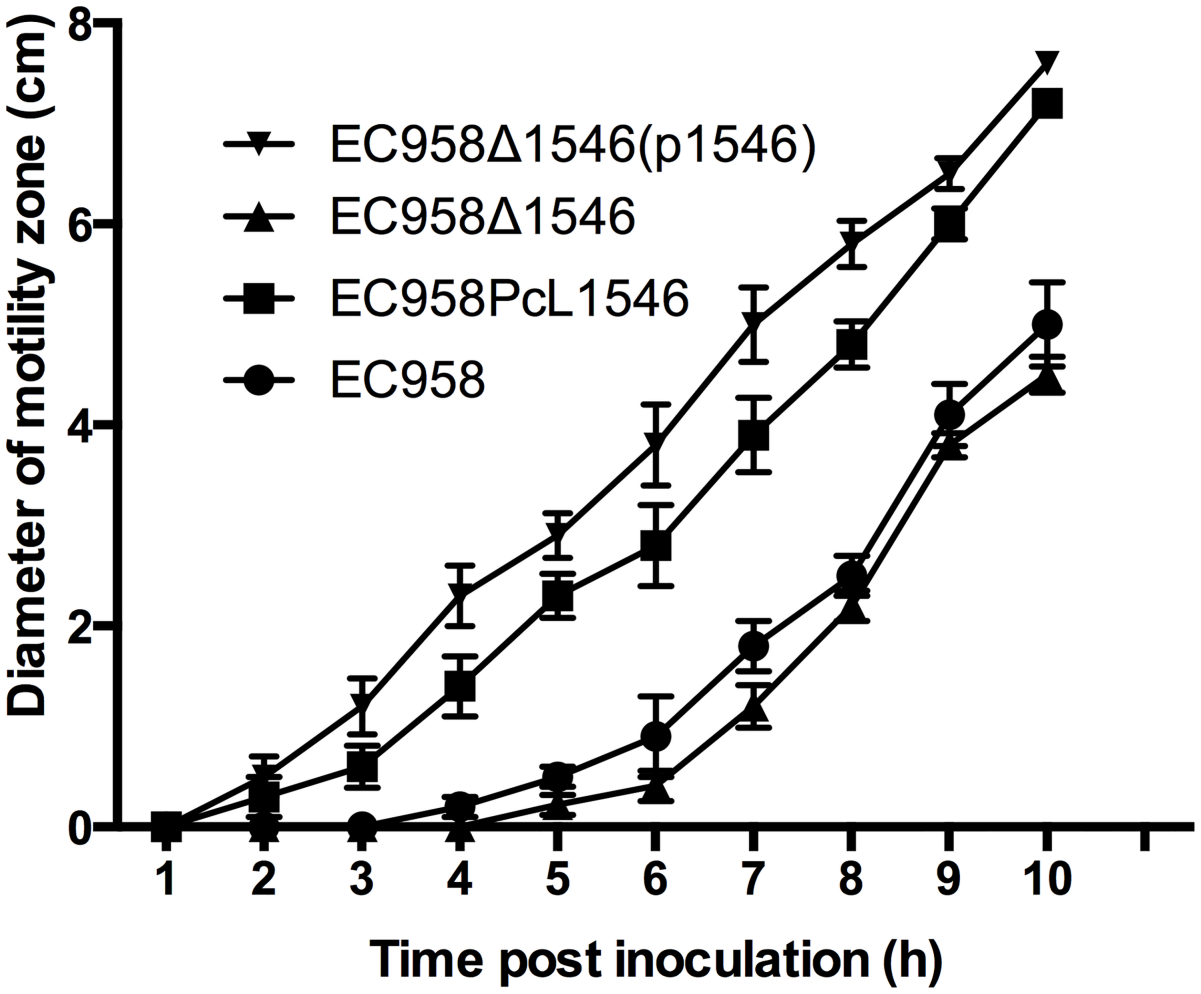
Overexpression of EC958_1546 leads to enhanced motility. Motility phenotype expressed as the diameter of the swimming zone per hour for EC958, EC958Δ1546, EC958PcL1546 and EC958Δ15469(p1546). The data represents the mean and standard deviation from three independent experiments.

The EC958_1546 gene is located within the phi4 prophage (EC958_Phi4: 1436674..1490889) and encodes a hypothetical phage protein. We hypothesized that like insertions in the IGR upstream of *flhDC*, the unidirectional miniTn*5* insertions in the IGR upstream of EC958_1546 enhanced its transcription and imparted a positive effect on motility. To investigate this further, we generated an isogenic EC958_1546 mutant (EC958Δ1546) by λ-red mediated homologous recombination. EC958Δ1546 motility was unchanged compared to wild-type EC958 (Fig 5), suggesting its deletion did not alter this phenotype. Next we cloned the EC958_1546 gene into the low copy number expression vector pSU2718 to generate plasmid p1546. Transformation of plasmid p1546 into EC958Δ1546 led to significantly enhanced motility (Fig 5). Finally, we constructed an EC958_1546 overexpressing strain by inserting a constitutive PcL promoter upstream of the chromosomal EC958_1546 gene (strain EC958PcL1546). The motility rate of EC958PcL1546 was also significantly higher than wild-type EC958 (Fig 5). Taken together, these data strongly support a role for the product of the EC958_1546 gene in enhancing motility in EC958.

### EC958_1546 overexpression enhances transcription of the *flhD* master regulator

**Fig 6:**
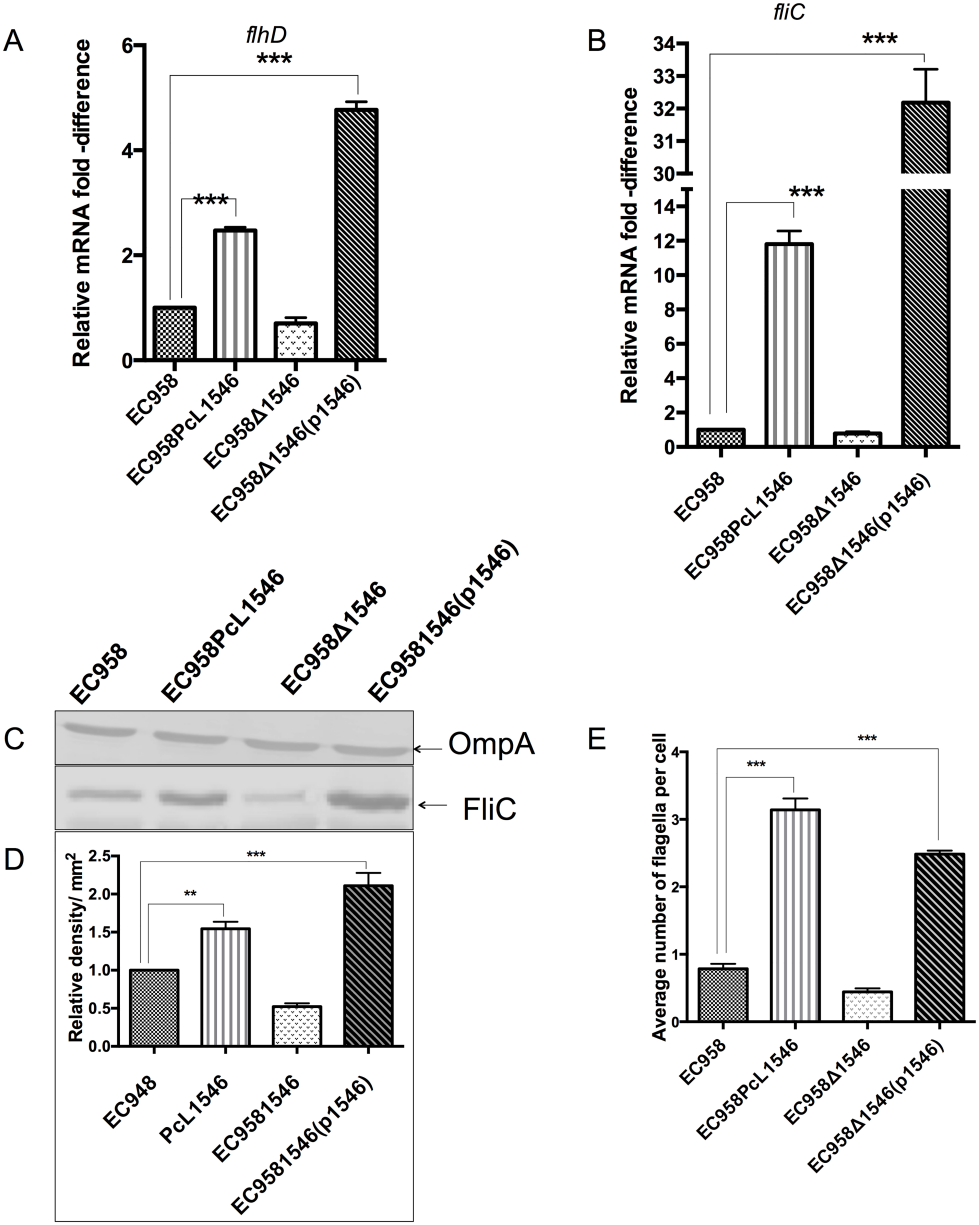
Overexpression of EC958_1546 leads to increased flagellar gene transcription, flagellin expression and flagella production. (A and B) Quantitative RT-PCR demonstrating the transcription level of (A) *flhD* and (B) *fliC* genes in wild-type, mutant and overexpression strains grown to mid-log phase. (C) Western blot analysis of cell lysates prepared from the same mid-log phase culture of each strain probed with an antibody against the H4 FliC flagellin protein (bottom panel) and OmpA (loading control; top panel). (D) Quantitative densitometry analysis of FliC protein production. (E) Average number of flagella counted from 200 cells for each strain following visualization by TEM; the mean and standard deviation from three independent experiments are shown. **P<0.01; ***P<0.001.

To investigate the mechanism by which EC958_1546 enhances motility, we used the same approach described above and examined *flhD* and *fliC* transcription by qRT-PCR, FliC expression by western blot analysis and flagella expression by TEM. Compared to wild-type EC958, the transcription of *flhD* was ~2-fold higher for EC958PcL1546 and ~4-fold higher for EC958Δ1546(p1546) (Fig 6A). Similarly, the transcription of *fliC* was also significantly increased (~11-fold for EC958PcL1546 and ~32-fold for EC958Δ1546(p1546); Fig 6B). Consistent with our motility analysis, no significant difference was observed in the transcription of *flhD* and *fliC in* EC958Δ1546 (Fig 6B). Overexpression of EC958_1546 also led to an increase in FliC expression (Fig 6C and 6D) and flagella production (Fig 6E, S3 Fig) compared to wild-type EC958. Thus, our data strongly support a mechanism whereby overexpression of EC958_1546 leads to enhanced transcription of the *flhDC* master regulator genes, resulting in increased flagella production and a hyper-motile phenotype.

### Overexpression of EC958_1546 also leads to hyper-motility of other UPEC strains

**Fig 7:**
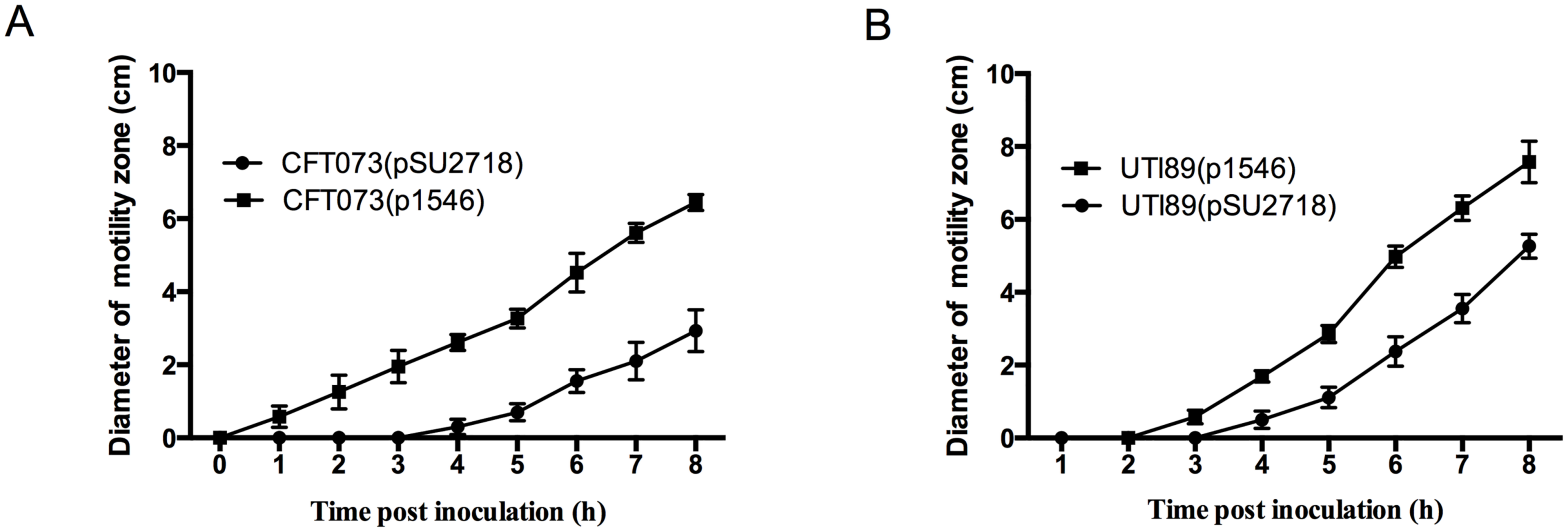
Overexpression of EC958_1546 in CFT073 and UTI89 leads to enhanced motility. Motility phenotype expressed as the diameter of the swimming zone per hour for (A) CFT073(pSU2718, vector control) and CFT073(p1546), and (B) UTI89(pSU2718, vector control) and UTI89(p1546). The data represents the mean and standard deviation from three independent experiments.

To extend our analysis on the function of EC958_1546, we also examined its overexpression in two other well-characterised UPEC strains, namely CFT073 and UTI89. Plasmid p1546 was transformed into both strains to generate CFT073(p1546) and UTI89(p1546), respectively. In both strains, overexpression of EC958_1546 led to increased motility compared to vector control strains (Fig 7), demonstrating that EC958_1546 can enhance motility in multiple UPEC strains (Fig 7).

## Discussion

The use of TraDIS to identify genes involved in motility represents a novel application for this high throughput forward genetic screen. We initially hypothesized that all mutants defective in swimming would be absent from the output pool, and thus that our screen would identify the complete flagella regulon of EC958. However, analysis of our TraDIS data did not reveal any genes that exhibited a significant reduction in insertion frequency in the output pool (compared to the input pool), suggesting that non-motile mutants are likely to be ‘carried’ by the wave of swimming cells in our assay. Indeed, this is consistent with the previous findings of Girgis *et al*. [39], who demonstrated a requirement for up to five rounds of selection and enrichment of swimming cells to identify genes essential for motility. Instead, our TraDIS analysis identified 30 genes associated with the enhanced motility of EC958. This included 12 genes encoding factors known to repress motility of *E*. *coli* K-12, four of which (*lrhA*, *ihfA*, *ydiV*, *lrp*) were confirmed in this study. The remaining genes represent novel factors that impact motility, and we focused our investigation on characterisation of the *mprA*, *hemK* and *yjeA* genes. Mutation of each of these genes in EC958 led to increased transcription of *flhD* and *fliC*, increased expression of the FliC flagellin, enhanced flagella synthesis and a hyper-motile phenotype. Importantly, all of these properties were restored to wild-type level upon complementation.

MprA (also known as EmrR) is a transcriptional regulator that belongs to the MarR family of winged helix DNA binding proteins, which control the expression of a range of bacterial genes involved in virulence, resistance to antibiotics, response to oxidative stresses and the catabolism of environmental aromatic compounds [58, 59]. In *E*. *coli*, the *mprA* gene is located in an operon together with the *ermAB* genes that encode a multidrug resistance pump [60, 61]. MprA represses transcription of *ermAB* by direct binding to its promoter region [62]. A recent study reported that MprA also controls UPEC capsule synthesis, and specific inhibitors of MprA prevented polysaccharide capsule production [63]. In this case, the effect of MprA on capsule production was indirect and most likely coordinated through a broader regulatory network. Here, we identified a new role for MprA in UPEC motility. Although the precise molecular mechanism by which MprA represses UPEC motility remains to be determined, our data suggest its effect is mediated at the transcriptional level, and could occur either directly by binding to the *flhDC* promoter region or indirectly by affecting the expression of other *flhDC* regulators. In this respect, we note that mutation of *ermAB* did not change the motility of EC958 (S4 Fig), ruling out an affect via altered expression of the ErmAB multidrug resistance pump. Among the other characterised MarR-like transcriptional regulators, PapX has also been shown to repress the motility of UPEC [35, 46]. PapX directly binds to the *flhDC* promoter and represses transcription, and its over-expression results in reduced flagellin production and decreased motility [35]. Consistent with these data, *papX* was also identified in our TraDIS screen. Taken together, our results provide strong evidence that in addition to PapX, MprA also affects UPEC motility.

HemK is a protein (N5)-glutamine methyltransferase that modulates the termination of release factors in ribosomal protein synthesis [64–66]. In *E*. *coli*, mutation of *hemK* causes defects in translational termination, leading to reduced growth rate and induction of the oxidative stress response [64, 66]. We also observed a significant growth defect for the EC958*hemK* mutant in comparison to wild-type EC958 and the complemented mutant EC958*hemK*(pHemK) (S5 Fig). Based on this knowledge, the observation that deletion of *hemK* leads to enhanced motility is difficult to understand. It is possible that the induction of multiple stresses in a *hemK* mutant background results in increased FlhDC expression. Indeed, FlhDC expression is responsive to a range of environmental stimuli (e.g. temperature, osmolarity and pH) [67].

YjeA (also known as PoxA) is a lysine 2,3-aminomutase that mediates post-translational modification of elongation factor-P (EF-P) [68–70]. EF-P is an essential component of bacterial protein synthesis and binds to ribosomes to facilitate peptide bond formation [71, 72]. In *E*. *coli*, the lysine residue 34 (Lys34) of EF-P is posttranslationally modified by YjeA, resulting in increased affinity of EF-P to the ribosome [73, 74] and prevention of ribosome stalling at polyproline stretches [73, 74]. EF-P Lys34 can also be modified by a second enzyme, YjeK [69]. Notably, both *yjeA* and *yjeK* were identified in our TraDIS motility screen (Table 1), suggesting that a defect in EF-P modification actually enhances the motility of EC958. In *Salmonella*, a contrasting motility phenotype for *yjeA* and *yjeK* mutants has been reported, with mutation of these genes leading to impaired motility [75, 76]. It is possible that these differences may be related to the relative abundance of flagella-related proteins that contain polyproline stretches between both organisms, although a direct comparison of flagella proteins in EC958 and the *Salmonella* strain UK-1 did not reveal any major differences (S3 Table). Alternatively, as observed for *hemK*, mutation of *yjeA* and *yjeK* may induce a stress response that leads to increased FlhDC expression. Overall, the precise mechanism by which mutation of *yjeA* results in enhanced motility requires further investigation.

Our TraDIS analysis also identified an additional 14 non-flagellar genes that, when mutated, led to enhanced motility of EC958. The function of these genes ranged across seven COG functional categories, including ‘cell wall/membrane/envelope biogenesis’ (2 genes), ‘mobilome: prophages, transposons’ (2 genes), Signal transduction (3 genes), aminoacid transport and metabolism (2 genes) and others (Table 1). Confirmation of the role of these genes in motility via the construction and characterisation of specific mutants is now required.

The use of a highly saturated mutant library in our TraDIS procedure also enabled the interrogation of miniTn*5* insertions within IGRs on the EC958 chromosome. In total, eight IGRs were identified that contained significantly more insertions in the output pool compared to the input pool, indicating insertions within these IGRs led to enhanced motility. Six of these IGRs were located upstream of CDS for genes known to repress motility, all of which were also identified in our screen. Close inspection of the Tn*5* insertions revealed their orientation was unidirectional and opposite to the direction of the downstream genes, consistent with the notion that the insertion disrupted transcription of the corresponding gene. The analysis also identified Tn*5* insertions in IGRs upstream of *flhDC* and EC958_1546. These Tn*5* insertions were also unidirectional, but instead orientated in the same direction as the respective downstream gene, which in both cases was devoid of Tn*5* insertions. We hypothesized that these Tn*5* insertions most likely resulted in enhanced transcription of the downstream gene(s); indeed this was confirmed by introducing the strong constitutive PcL promoter upstream of both genes, which resulted in enhanced motility. Thus, our approach has revealed a novel application of TraDIS to identify genes that enhance a specific phenotype when their transcription is increased.

EC958_1546 encodes a hypothetical phage protein predicted to be 617 amino acids in length. EC958_1546 displays 58% identity over 326 amino acids to NanS, an N-acetylneuraminic acid deacetylase that catalyses the hydrolysis of the 9-O-acetyl group of 9-O-acetyl-N-acetylneuraminate, an alternative sialic acid commonly found in mammalian host mucosal sites such as the human intestine [77–79]. We speculate that over-expression of the EC958_1546 protein may enhance motility via an altered chemotactic response, however this remains to be experimentally proven. Interestingly, there are three additional genes on the EC558 chromosome that display similarity to EC958_1546, namely EC958_1029 (82.7% amino acid identity over the whole protein), EC958_3294 (57.4% amino acid identity over 317 amino acids) and EC958_0037 (58.4% amino acid identity over 319 amino acids) (S6 Fig). None of these three genes were identified in our TraDIS screen. Furthermore, PCR amplification, cloning and overexpression of these genes in EC958 did not alter motility (S7 Fig), confirming the specific affect of EC958_1546 on this phenotype. We also showed that over-expression of EC958_1546 in two other UPEC strains could also invoke an enhanced motility phenotype, demonstrating the affect is not strain specific. In this respect, an Stx-phage-encoded protein (933Wp42) from enterohemorrhagic *E*. *coli* that possesses 53% amino acid identity with EC958_1546 has been shown to have esterase activity [78], and other phage-encoded variants of *nanS* have been described [80]. Thus, it is possible that the over-expression of other phage proteins with the capacity to degrade different carbon sources could also impact motility.

Overall, this study demonstrates the application of TraDIS to identify novel genes associated with enhanced motility. A better understanding of the mechanisms by which many of the genes identified enhance motility is now required.

## Materials and methods

### Bacterial strains and growth conditions

All strains and plasmids used in this study are listed in Table 3. Strains were routinely cultured at 37°C on solid or in liquid Lysogeny Broth (LB) medium supplemented with the appropriate antibiotics (chloramphenicol 30 μg/ml or gentamicin 20 μg/ml) unless indicated otherwise. Where necessary, gene expression was induced with 1mM isopropyl β-D-1-thiogalactopyranoside (IPTG).

**Table 3:** Bacterial strains and plasmids used in this study.

Strain/Plasmid	*Relevant characteristics*	*Reference*
EC958	ST131 reference strain; H4	[81]
UTI89	UPEC reference strain	[82]
CFT073	UPEC reference strain	[83]
EC958*lrhA*	EC958 *lrhA*::cam; Cam^r^	This study
EC958*ihfA*	EC958 *ihfA*::cam; Cam^r^	This study
EC958*ydiV*	EC958 *ydiV*::cam; Cam^r^	This study
EC958*lrp*	EC958 *lrp*::cam; Cam^r^	This study
EC958*mprA*	EC958 *mprA*::cam; Cam^r^	This study
EC958*hemK*	EC958 *hemK*::cam; Cam^r^	This study
EC958*yjeA*	EC958 *yjeA*::cam; Cam^r^	This study
EC958Δ1546	EC958 1546::cam; Cam^r^	This study
EC958PcL*flhDC*	EC958PcLflhDC; constitutively expressed *flhDC*	This study
EC958PcL1546	EC958PcL*1546*; constitutively expressed 1546	This study
pMprA	*EC958 mprA* gene in BamHI-XbaI digested pSU2718G	This study
pHemK	*EC958hemK* gene in BamHI-XbaI digested pSU2718G	This study
pYjeA	*EC958 yjeA* gene in BamHI-XbaI digested pSU2718G	This study
p1546	EC958_1546 gene in XbaI-Sac1 digested pSU2718	This study
p1029	EC958_1029 gene in Xba1-Sac1 digested pSU2718	This study
p3294	EC958_3294 gene in Xba1-Sac1 digested pSU2718	This study
P0037	EC958_0037 gene in Xba1-Sac1 digested pSU2718	This study
pKD3	Template plasmid for *cam* gene amplification	[84]
pKOBEG-Gent	λ-Red plasmid, Gent^r^	[85]
pCP20-Gent	FLP-recombinase plasmid	[86]
pSU2718	Cloning vector, Cam^r^	[87]

### Molecular methods

DNA purification, PCR and Sanger DNA sequencing was performed as previously described [88]. Targeted mutations were generated using a modified λ-Red recombineering method [81, 84]. A list of primers used in this study is provided in S2 Table. In brief, the final PCR products were generated by a 3-way PCR that resulted in amplification of the chloramphenicol resistance gene cassette flanked by 500-bp homologous regions matching the target gene to be mutated. The PCR products were electroporated into EC958 harbouring pKOBEG-Gent. Mutants were selected by growth in the presence of chloramphenicol and confirmed by sequencing. Complementation was performed by cloning the gene of interest into pSU2718 [87] or pSU2718G. The resultant plasmid was then transformed into the respective mutant and gene expression was induced using 1 mM IPTG.

### Screening assay for identification of mutants with enhanced motility

Approximately 1x10^7^ cells from a previously constructed miniTn*5* mutant library of EC958 (input pool; [43]) were inoculated into the center of each of 20 LB soft agar plates (80 mm diameter) and incubated for 10 hours at 37°C. Motile cells were recovered by extracting the LB agar at a distance of 30 mm from the point of inoculation (the edge of the swimming zone; output pool). Approximately 5g of soft agar (plus motile cells) was collected from each plate and vigorously mixed with LB broth to achieve a suspension of 1g agar/ml. Five ml of this mixture was drawn from each tube (n = 20) and pooled. The pooled mixture was centrifuged at 6000 rpm for 10 min at room temperature to separate the bacterial pellet from soft agar. This centrifugation step produced a tight bacterial pellet surrounded by a loose mass of soft agar and a layer of supernatant. The agar and supernatant was removed, and the pellet was resuspended in LB to an OD_600_ of 1.8; genomic DNA was extracted from 5 ml of this suspension using the Qiagen genomic DNA purification kit. DNA from the input pool was extracted in the same manner from the EC958 mutant library [43].The screening assays were performed in duplicates.

### Multiplexed TraDIS

TraDIS was performed essentially as previously described [43], but with some modifications for adaptation to the MiSeq platform [89]. Briefly, 50 ng of genomic DNA from each sample (2 biological replicates of input and output pools, respectively) was fragmented and tagged with adapter sequence via one enzymatic reaction (tagmentation). Following tagmentation, DNA was purified using Zymo DNA Clean & Concentrator^™^ kit (Zymo Research). The PCR enrichment step was run using index primer 1 (one index per sample) and a custom transposon specific primer 4844 (5’-AATGATACGGCGACCACCGAGATCTACACTAGATCGCaacttcggaataggaactaagg-3’) to enrich for transposon insertion sites and allow for multiplexing sequencing; the thermocycler program is 72°C for 3 minutes, 98°C for 30 seconds followed by 22 cycles of 98°C for 10 seconds, 63°C for 30 seconds and 72°C for 1 minute. Each library was purified using Agencourt^®^ Ampure^®^ XP magnetic beads. Verification and quantification of resulting libraries were calculated using a Qubit^®^ 2.0 Fluorometer, 2100 Bioanalyser (Agilent Technologies) and qPCR (KAPA Biosciences). All libraries were pooled in equimolar to a final concentration of 3.2nM and submitted for sequencing on the MiSeq platform at the Queensland Centre for Medical Genomics (Institute for Molecular Bioscience, The University of Queensland). The MiSeq sequencer was loaded with 12 pM of pooled library with 5% PhiX spike-in and sequenced (single-end, 101 cycles) using a mixture of standard Illumina sequencing primer and Tn*5*-specific sequencing primer 4845 (5’- actaaggaggatattcatatggaccatggctaattcccatgtcAGATGTG-3’). A total of two MiSeq runs were performed to achieve sufficient read depth for analysis. All experiments were performed in duplicate. The TraDIS sequence data from this study was deposited on the Sequence Read Archive (SRA) under the Bio Project number PRJNA339173 (http://www.ncbi.nlm.nih.gov/sra/SRP082245).

### Analysis of TraDIS data

The raw, de-multiplexed fastq files from both MiSeq runs were combined and filtered to capture reads containing the 12-bp Tn*5*-specific barcode (5'-TATAAGAGACAG-3'), allowing for 2 mismatches (fastx_barcode_splitter.pl, FASTX-Toolkit v.0.0.13). These reads were trimmed to remove the 12-bp barcode and 58-bp at the 3' end (fastx_trimmer, FASTX-Toolkit v.0.0.13), resulting in high quality sequence reads of 30-bp in length that were mapped to the EC958 chromosome (gb|HG941718) by Maq version 0.7.1 [90]. Subsequent analysis steps were carried out using an in-house Perl script as previously described [43] to calculate the number of unique insertion sites and the read count at each site for every gene and IGR.

### Statistical analysis

EC958 genes and IGRs associated with enhanced motility were identified by comparing their relative read abundance in the input and output pools using the Bioconductor package edgeR [91] as previously described [43]. Briefly, the read counts from each sample were loaded into the edgeR package (version 2.6.12) using the R environment (version 2.15.1). The composition bias in each sequence library was normalized using the trimmed mean of M value (TMM) method [92]. The quantile-adjusted conditional maximum likelihood (qCML) for negative binomial models was then used to estimate dispersions (biological variation between replicates) and to perform exact tests for determining genes and IGRs with significantly lower read counts in the input pools compared to the output pools as previously described [93, 94]. Stringent criteria of log fold- change (logFC) ≥5 and false discovery rate ≤ 0.001 were used to define a list of the most significant genes for further investigation by phenotypic assays. All other experimental data were analyzed using unpaired Students t-test and *P*-values ≤ 0.05 were considered to be statistically significant.

### Motility assay

To evaluate motility, 6 μl of an overnight culture prepared in LB broth was spotted onto the centre or the edge of a freshly prepared 0.25% LB Bacto-agar plate (*n* = 3), supplemented with the appropriate inducer and/or antibiotic. Plates were incubated at 37°C in a humid environment (a closed box containing a dish of water) and the rate of motility was determined by measuring the diameter of the motility zone over time.

### qRT-PCR

qRT-PCR was carried out essentially as previously described [14]. In brief, exponentially growing cells (OD_600_ 0.6) were stabilized with two-volumes of RNAprotect Bacteria Reagent (Qiagen) prior to RNA extraction using the RNeasy Mini Kit (Qiagen) followed by on-column DNase digestion. First-strand cDNA synthesis was performed using SuperScript^®^ III First-Strand Synthesis System (Invitrogen) as per manufacturer’s recommendation. Real-time PCR was performed using SYBR^®^ Green PCR Master Mix (Applied Biosystems) on the ViiA^™^ 7 Real-Time PCR System (Applied Biosystems) using the following primers: *flhD*, primers 5613 (5′-acttgcacagcgtctgattg) and 5614 (5′-agcttaaccatttgcggaag); *fliC*, primers 5683 (5′-caccaacctgaacaacacca) and 5684 (5′-gcacggcgaatatccagttg). Transcript levels of each gene were normalized to *gapA* as the endogenous gene control (primers 820, 5′-ggtgcgaagaaagtggttatgac and 821, 5′-ggccagcatatttgtcgaagttag). Gene expression levels were determined using the 2^-ΔΔCT^ method with relative fold-difference expressed against EC958.

### Protein preparation and western blotting

Whole cells lysates were prepared by pelleting 1 ml of an overnight culture diluted to an optical density at 600nm (OD_600_) of 1.0, and resuspending in 50 μl of distilled water plus 50 μl of 2x SDS loading buffer. SDS PAGE and transfer of proteins to a PVDF membrane for western blotting was performed as previously described [53]. Monospecific antiserum against H4 flagellin was purchased from the Statens Serum Institute, Denmark. OmpA antiserum was purchased from the Antibody Research Corporation, USA (item #111120). Primary antibodies were detected with commercially purchased alkaline phosphatase-conjugated anti-rabbit antibody (Sigma Aldrich). SIGMAFAST^™^BCIP^®^/NBT (Sigma Aldrich) was used as substrate for detection.

## Supporting information

S1 Fig TEM analysis demonstrating flagella expression for representative EC958 wild-type, mutant and complemented strains. (TIF)

S2 Fig Overexpression of the *flhDC* master regulator genes in EC958 leads to enhanced motility. Left panel, motility phenotype expressed as the diameter of the swimming zone per hour for EC958 and EC958PcL*flhDC*. The data represents the mean and standard deviation from three independent experiments. Right panel, western blot analysis of cell lysates prepared from mid-log phase cultures of EC958 and EC958PcL*flhDC* probed with an antibody against the H4 FliC flagellin protein (top panel) and OmpA (loading control; bottom panel).
(TIF)

S3 Fig TEM analysis demonstrating flagella expression for representative EC958, EC958Δ1546, EC958PcL1546 and EC958Δ15469(p1546) strains. (TIF)

S4 Fig Motility phenotype of EC958 and EC958*emrAB* strains. Motility is expressed as the diameter of the swimming zone per hour for EC958 and EC958*emrAB*. The data represents the mean and standard deviation from three independent experiments.
(TIF)

S5 Fig Growth of EC958, EC958*hemK* and the complemented mutant EC958*hemK*(pHemK). EC958*hemK* displayed a reduced growth rate compared to the wild-type and complemented strains.
(TIF)

S6 Fig Amino acid alignment of the translated sequences for EC958_1546, EC958_1029, EC958_3294 and EC958_0037. Sequence alignments were performed using CLC main workbench 7.0.2. Residues identical to EC958_1546 are indicated by dots; gaps are indicated by dashed lines.
(TIF)

S7 Fig Motility phenotype of EC958(p1546), EC958(p1029), EC958(p3294), EC958(p0037) and EC958(pSU2718). Motility is expressed as the diameter of the swimming zone per hour for each strain. The data represents the mean and standard deviation from three independent experiments.
(TIF)

S1 Table Primers used in this study. (XLSX)

S2 Table Summary of sequencing and mapping results of TraDIS runs. (XLSX)

S3 Table Frequency of Proline residues in flagella-related proteins of EC958 and *Salmonella enterica* serovar Typhimurium strain UK-1. (XLSX)
