# Supplementary figures and images for: Novel genes associated with enhanced motility of Escherichia coli ST131
Source: PLoS One. 2017 May 10;12(5):e0176290. doi: 10.1371/journal.pone.0176290 (PMC5425062; doi:10.1371/journal.pone.0176290)

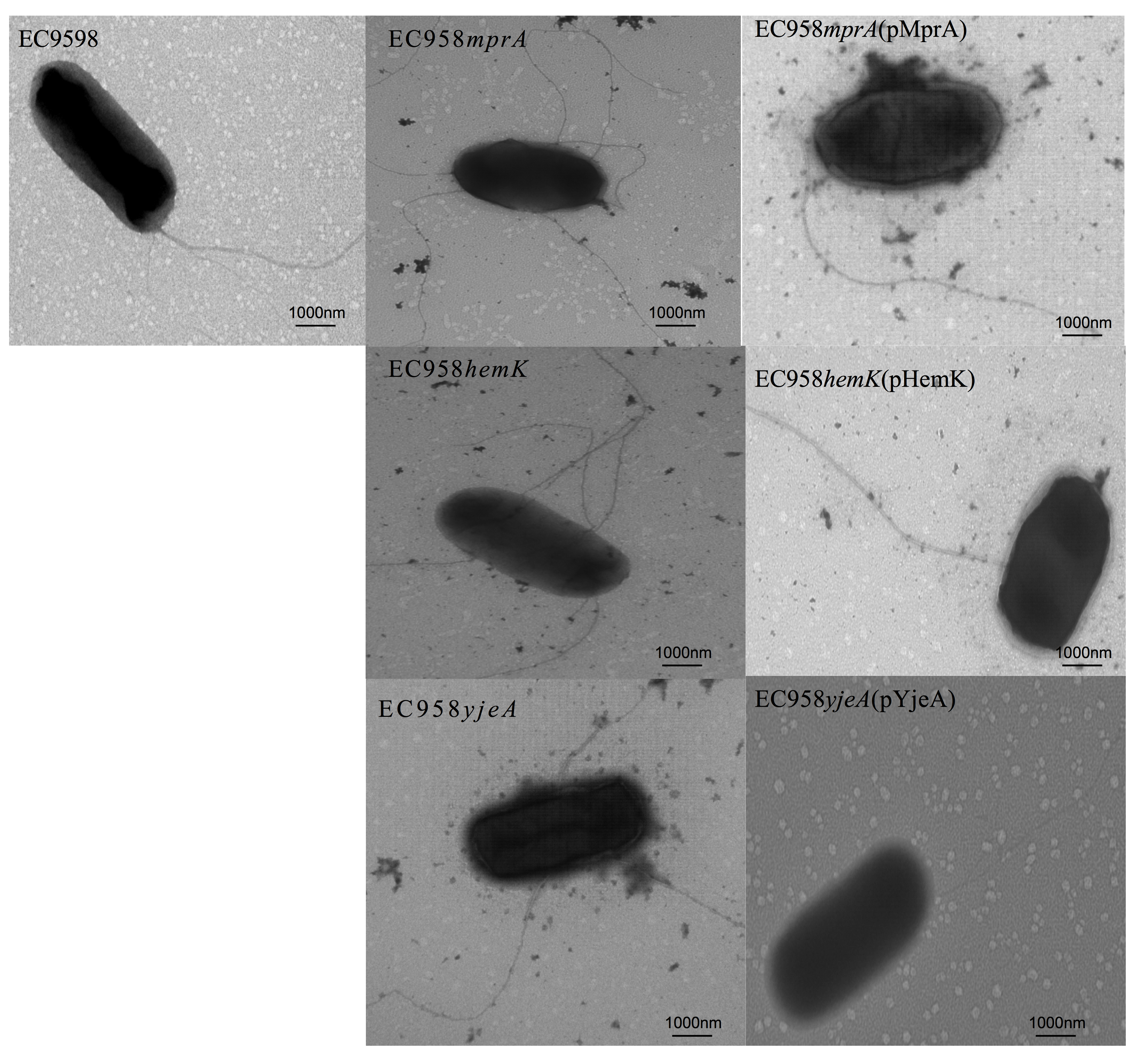

Supplement: S1 Fig — (TIF) [file pone.0176290.s001.tif]

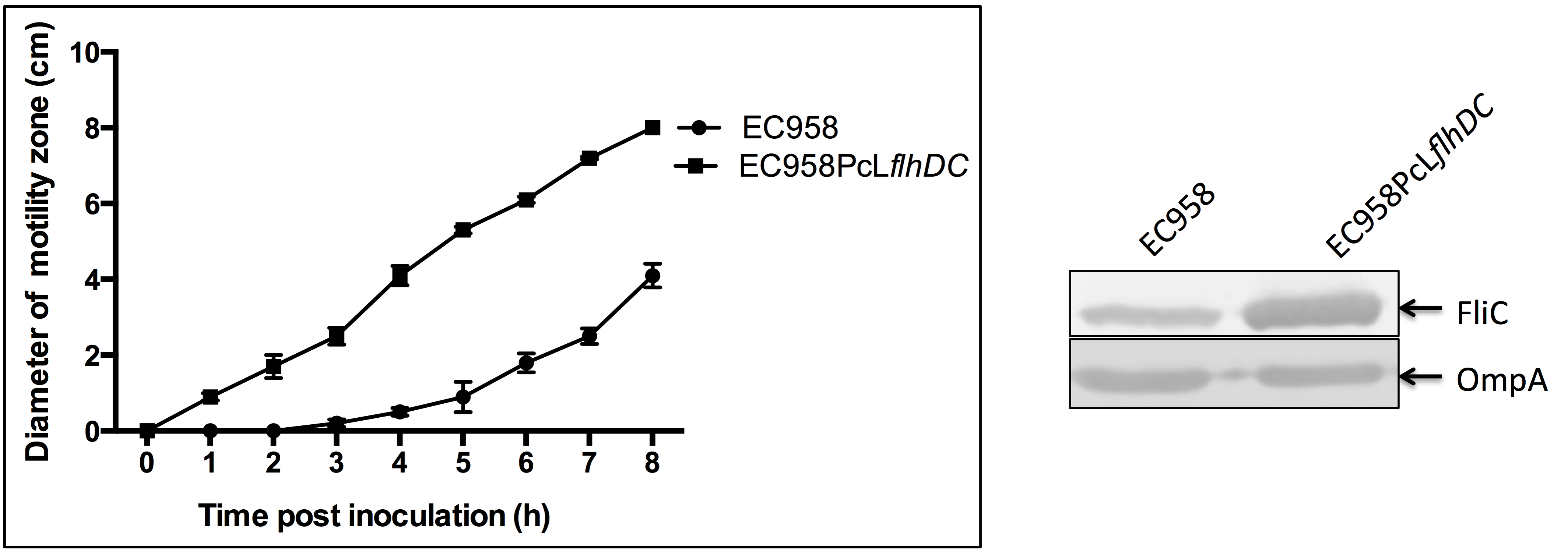

Supplement: S2 Fig — Left panel, motility phenotype expressed as the diameter of the swimming zone per hour for EC958 and EC958PcLflhDC. The data represents the mean and standard deviation from three independent experiments. Right panel, western blot analysis of cell lysates prepared from mid-log phase cultures of EC958 and EC958PcLflhDC probed with an antibody against the H4 FliC flagellin protein (top panel) and OmpA (loading control; bottom panel). (TIF) [file pone.0176290.s002.tif]

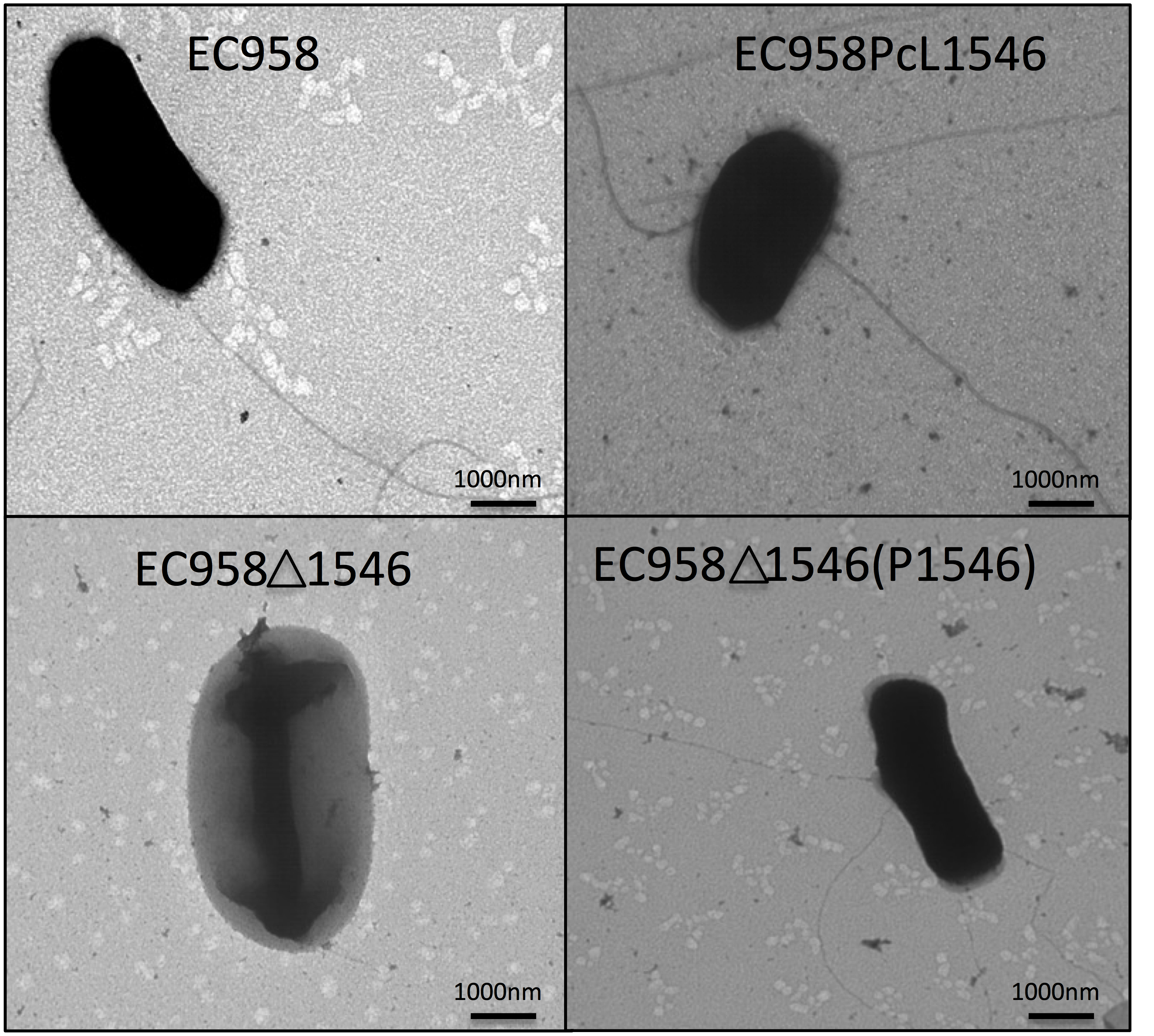

Supplement: S3 Fig — (TIF) [file pone.0176290.s003.tif]

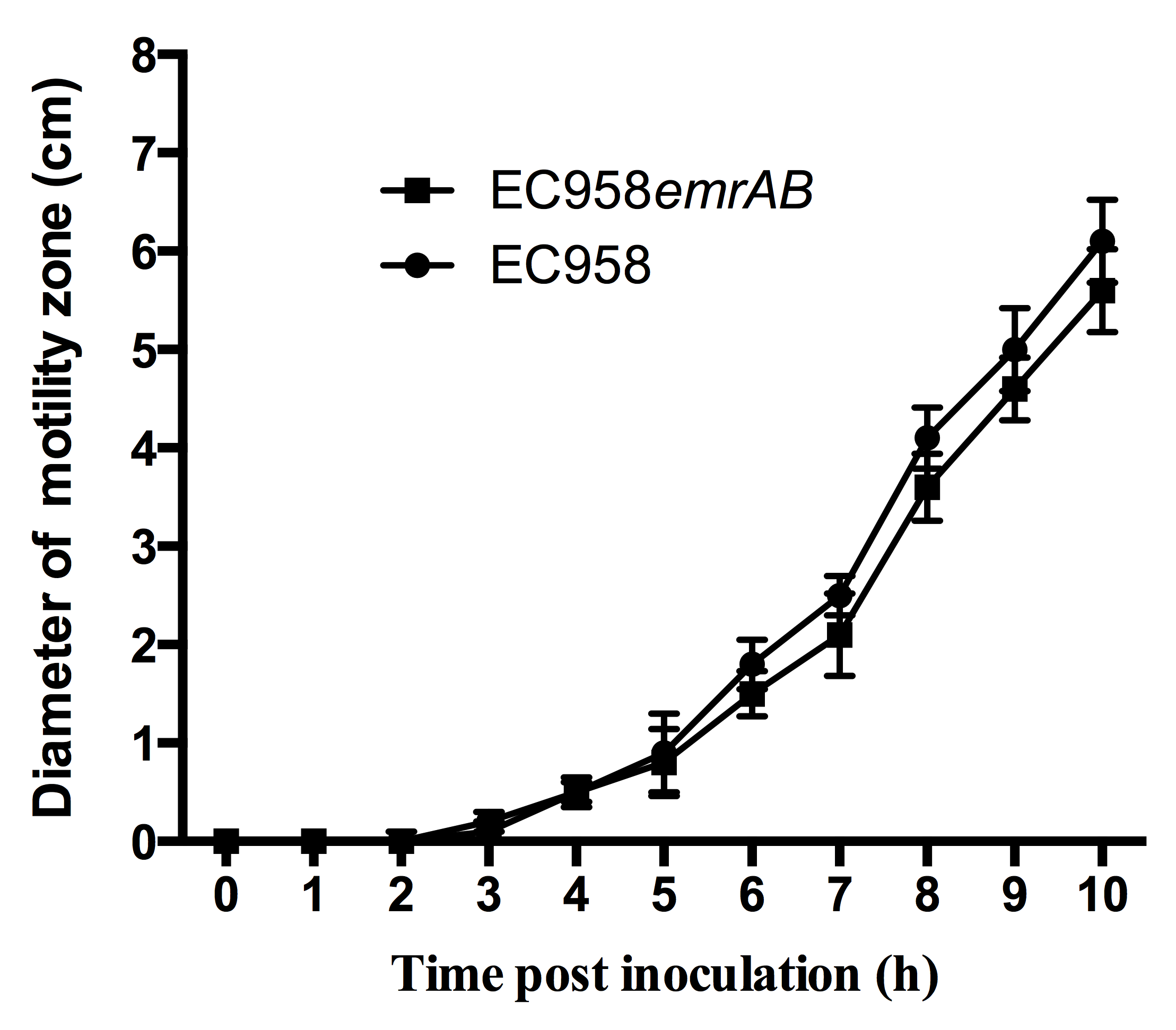

Supplement: S4 Fig — Motility is expressed as the diameter of the swimming zone per hour for EC958 and EC958emrAB. The data represents the mean and standard deviation from three independent experiments. (TIF) [file pone.0176290.s004.tif]

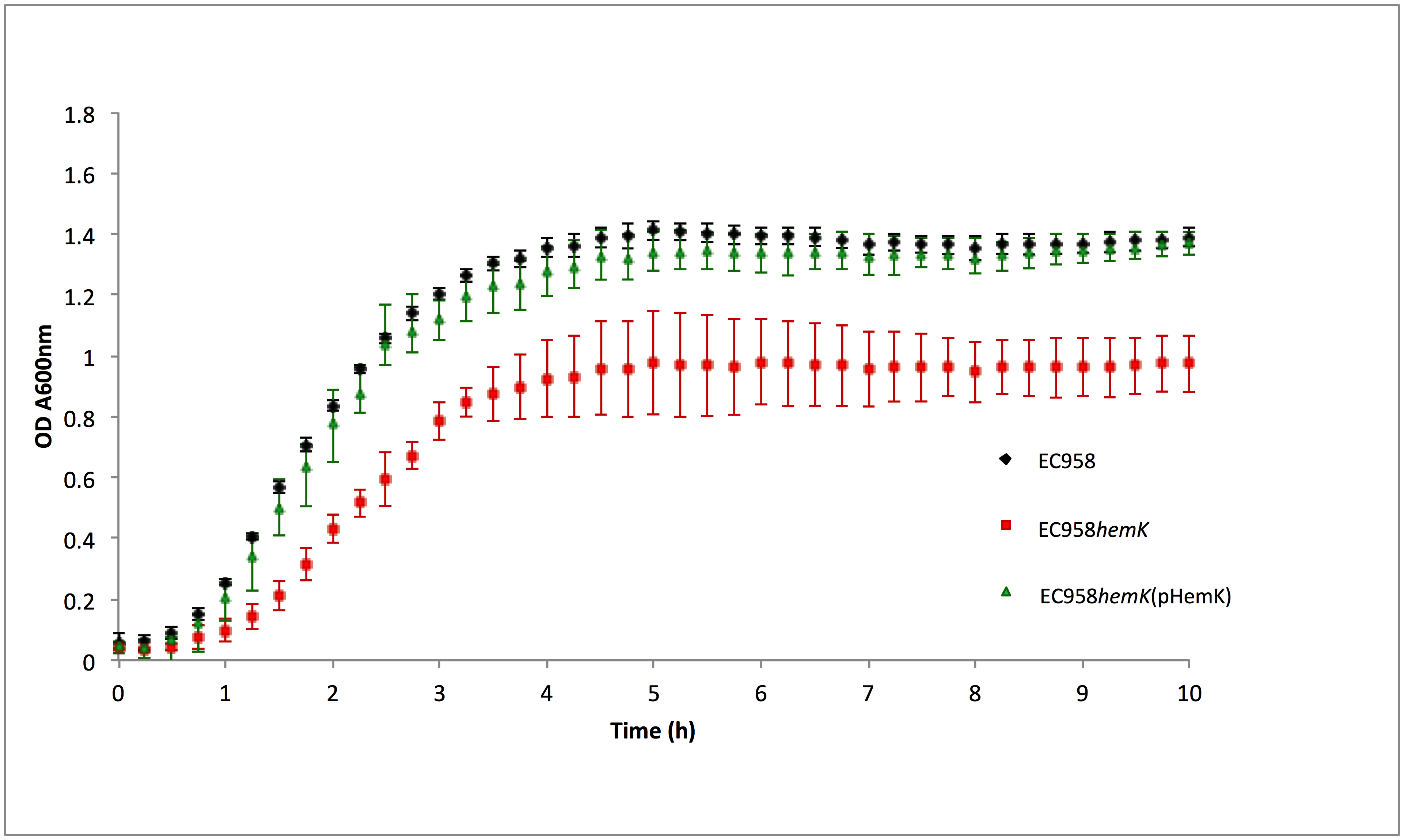

Supplement: S5 Fig — EC958hemK displayed a reduced growth rate compared to the wild-type and complemented strains. (TIF) [file pone.0176290.s005.tif]

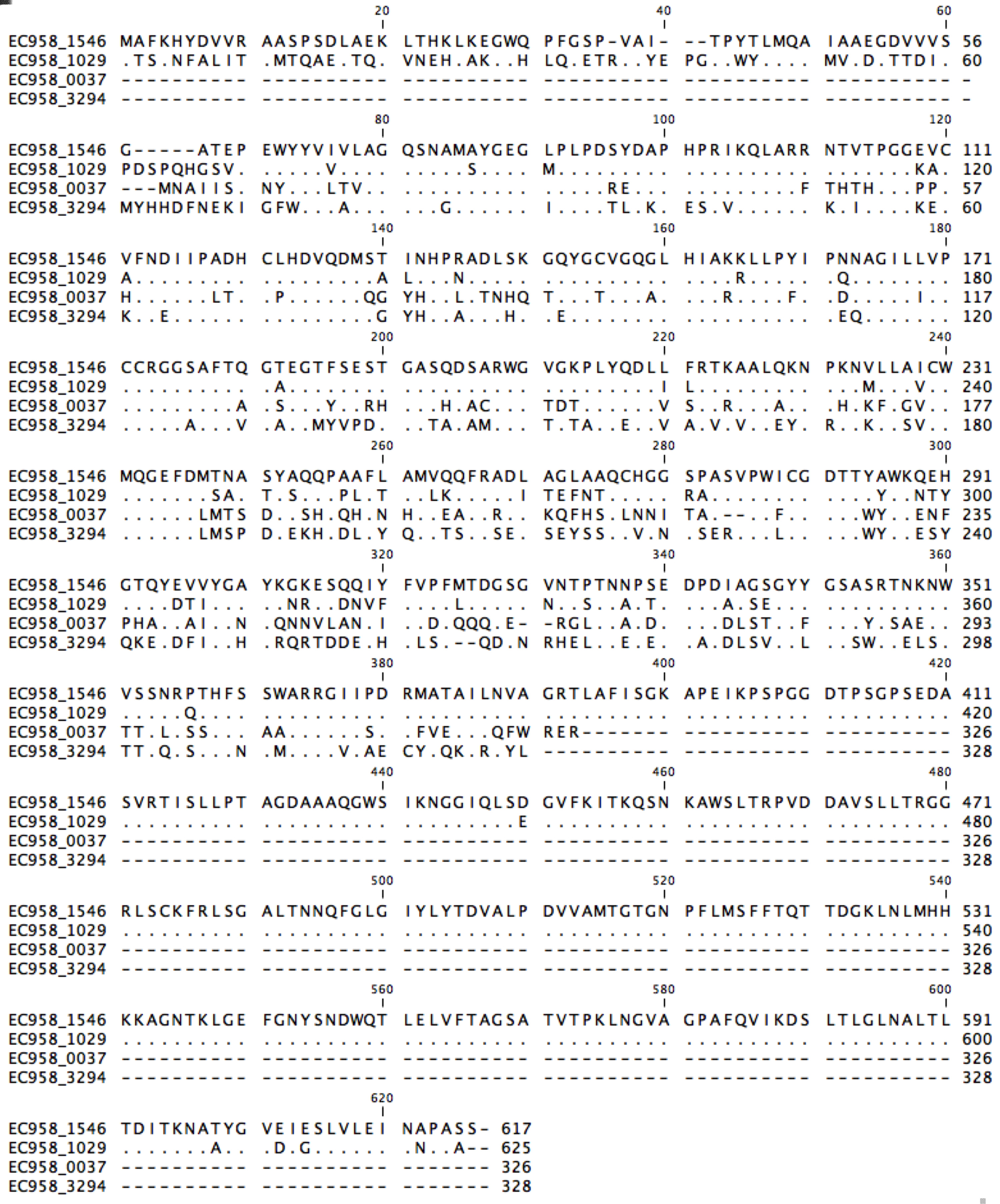

Supplement: S6 Fig — Sequence alignments were performed using CLC main workbench 7.0.2. Residues identical to EC958_1546 are indicated by dots; gaps are indicated by dashed lines. (TIF) [file pone.0176290.s006.tif]

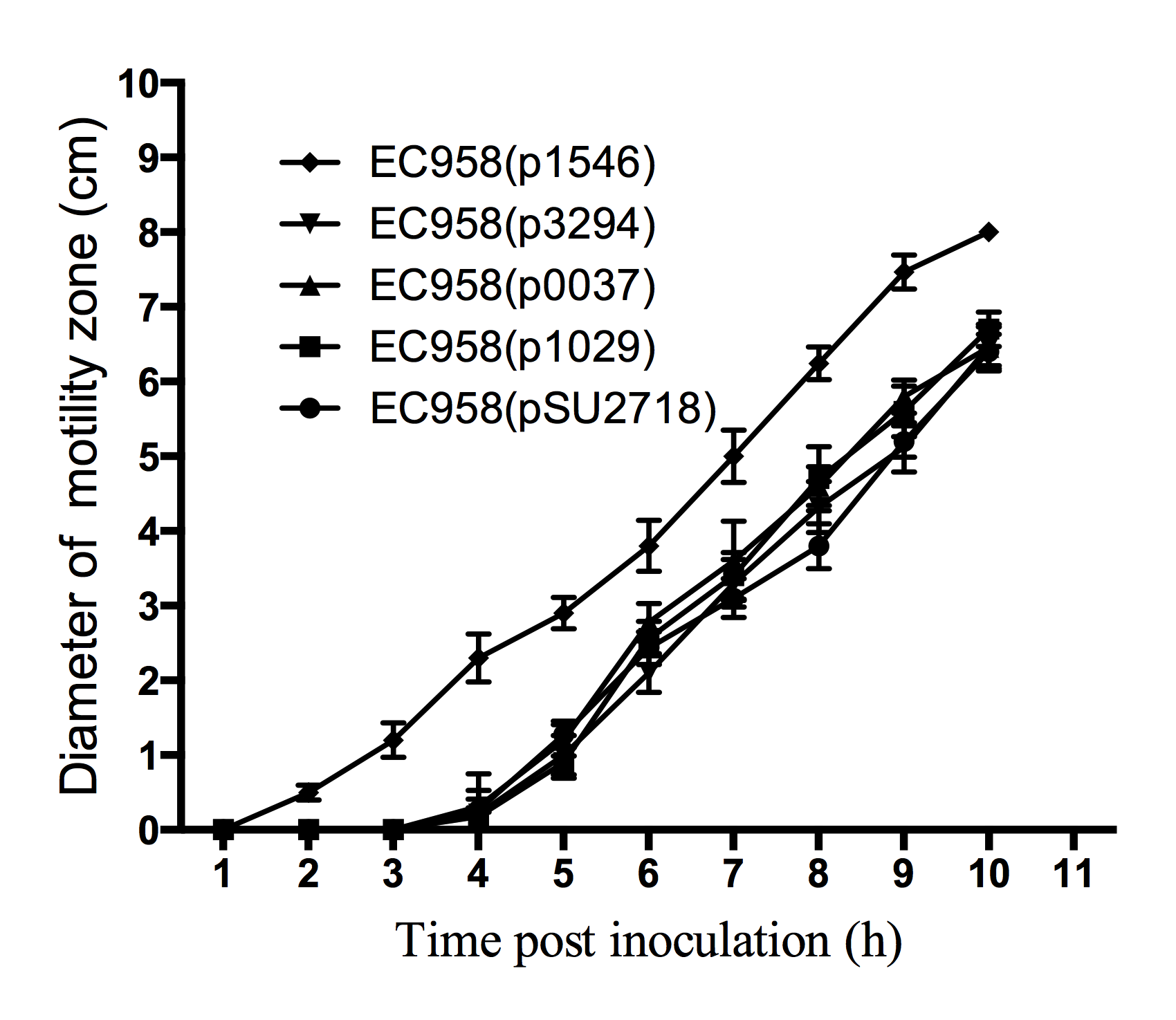

Supplement: S7 Fig — Motility is expressed as the diameter of the swimming zone per hour for each strain. The data represents the mean and standard deviation from three independent experiments. (TIF) [file pone.0176290.s007.tif]
